# Medication-related burden and associated factors among diabetes mellitus patients at Felege Hiwot Comprehensive Specialized Hospital in northwest Ethiopia

**DOI:** 10.3389/fcdhc.2022.977216

**Published:** 2022-09-09

**Authors:** Abaynesh Fentahun Bekalu, Melaku Kindie Yenit, Masho Tigabe Tekile, Mequanent Kassa Birarra

**Affiliations:** ^1^ Department of Clinical Pharmacy, School of Pharmacy, College of Medicine and Health Sciences, University of Gondar, Gondar, Ethiopia; ^2^ Department of Epidemiology and Biostatistics, College of Medicine and Health Sciences, University of Gondar, Gondar, Ethiopia

**Keywords:** medication-related burden, adherence, perceived global burden, diabetes mellitus, Ethiopia

## Abstract

**Background:**

Evaluating the medicine burden from the patients’ perspective is essential for getting good health outcomes of diabetes mellitus (DM) management. However, data are limited regarding this sensitive area. Thus, the study was aimed to determine the medication-related burden (MRB) and associated factors among DM patients at Felege Hiwot Comprehensive Specialized Hospital (FHCSH) in northwest Ethiopia.

**Methods:**

A cross-sectional study was conducted on 423 systematically selected DM patients attending the DM clinic of FHCSH from June to August 2020. The medication-related burden was measured by using the Living with Medicines Questionnaire version 3 (LMQ-3). Multiple linear regression was used to identify factors associated with medication-related burden and reported with 95% confidence interval (CI). *p*-value <0.05 was considered as statistically significant to declare an association.

**Results:**

The mean LMQ-3 score was 126.52 ( ± 17.39). The majority of the participants experienced moderate (58.9%, 95% CI: 53.9–63.7) to high (26.2%, 95% CI: 22.5–30.0) degrees of medication burden. Nearly half (44.9%, 95% CI: 39.9–49.7) of the participants were non-adherent to their prescribed medications. VAS score (*B* = 12.773, *p* = 0.001), ARMS score (*B* = 8.505, *p* = 0.001), and fasting blood glucose (FBS) on visit (*B* = 5.858, *p* = 0.003) were significantly associated with high medication-related burden.

**Conclusion:**

A significant number of patients suffered from high medication-related burden and non-adherence to long-term medicine. Therefore, multidimensional intervention to decrease MRB and to upgrade adherence is required to increase patients’ quality of life.

## Introduction

Diabetes mellitus (DM) is a global public health problem that causes significant morbidity and mortality (1, 2). Furthermore, DM and its complications are a reason for an increased risk of hospitalization, length of hospital stay, healthcare cost, and reduced health-related quality of life (2–6). In 2019, the estimated global prevalence of DM was 9.3% (463 million people), which is expected to rise to 10.9% (700 million) by 2045. In Ethiopia, according to the International Diabetes Federation 2019 report, the prevalence of DM was 4.3% (7). A few other studies in Ethiopia also reported that the prevalence of DM ranges from 1.9% to 12.2% (3, 8–12). Based on the literature search, studies regarding the prevalence of DM in the study area were not conducted. However, a single study on medication non-adherence and associated factors among diabetic patients reported that in northwest Ethiopia specifically at Felege Hiwot Comprehensive Specialized Hospital (FHCSH) approximately 2,484 DM patients were registered for follow-up and receiving diabetic care (13).

Medication-related burden (MRB) is a recent concept concerned with the negative experiences resulting from the process of undertaking treatment. It is one aspect of treatment burden, and it includes not only the burden of the medication but also all types of healthcare intervention and patient’s perspective toward MRB (14–16). Medication-related burden can lead to non-adherence and poor clinical outcomes, as well as affecting patient satisfaction, psychological well-being, social functioning, and quality of life. It can lead to poor quality of life as patients spend more of their time, energy, and resources on staying well because they experience burden not only from their illness but also from their ever-expanding healthcare regimens. Burdened patients may struggle with adhering to prescribed medications and treatment care, but the burden may influence adherence to treatment and patients’ health. Patients with multimorbidity and an excessive burden of treatment may not adhere to the prescribed medication (17–19).

Previously conducted studies described that MRB is affected by a variety of sociodemographic, clinical, and treatment-related factors. The significant predictors of high MRB include being male, older age, unemployed, polypharmacy, number of chronic conditions, the severity of the chronic condition, visual analog scale (VAS)-burden scores, needing support, high dosing frequency, paying prescription charge, Adherence to Refills and Medication Scale (ARMS) score, duration of DM, marital status, and presence of hypertension (16, 20, 21). A study done in Qatar reported that variables such as ARMS score, duration of DM, marital status, employment status, and presence of hypertension were significantly associated with MRB (16).

Patient-reported experience measures and patient-reported outcome measures have a pivotal role in helping patients know how they feel about their own experiences and outcomes of care, including the benefits and risks of treatment (22–24).

Previous studies explained that a high burden of treatment is a reason for increased hospitalization, cognitive impairment, drug interactions, physical side effects, increased healthcare resource utilization, and higher mortality rate. Furthermore, these studies suggest that exploring new interventions to reduce the burden of treatment, ultimately moving toward minimally disruptive medicine, is necessary (22, 23, 25–30). To help patients move toward minimally disruptive medicine, the identification and targeting of risk factors for high medication-related burden is the starting point. Evidence showed that DM is one of the most common chronic illnesses associated with micro- and macrovascular complications that might result in poor clinical outcomes and increased morbidity as well as mortality. Additionally, as DM is associated with a number of comorbidities such as hypertension and dyslipidemia, DM patients might be burdened by medicines (1–4). Despite the growing burden and economic impact of treatment burden in developing countries including Ethiopia, few studies have been conducted globally and most of them were carried out in the developed world. Based on the literature search, studies regarding this sensitive issue are lacking in Ethiopia including studies conducted in this specific country. Therefore, the study was aimed to determine the MRB and associated factors among DM patients at FHCSH in northwest Ethiopia.

## Methods and materials

### Study design, setting, and period

A cross-sectional study was conducted at FHCSH from June to August 2020, which is found in Bahir Dar City, northwest of Ethiopia. Bahir Dar is the capital city of Amhara regional state which is 564 km away from Addis Ababa. The hospital is expected to serve more than seven million people in its catchment area. It has 400 beds and 15 adult outpatient departments, one of which serves as a referral and follow-up clinic for patients with chronic diseases. The DM clinic is situated inside the outpatient department, and a large number of patients attend the follow-up clinic. Based on a previous study, around 2,484 DM patients were registered for follow-up in the clinic (31).

### Population

All adult DM patients attending the outpatient clinic of FHCSH were taken as the source population, whereas adult DM patients attending the outpatient clinic of FHCSH during the study period were considered as the study population. Adults (≥18 years) with a diagnosis of DM for at least 3 months prior to the study, with or without comorbidities, were included. However, DM patients with mental disabilities, any speech impairment, and incomplete charts were excluded.

### Sample size determination and sampling technique

A single population proportion formula [*n* = (*Z α*/2)^2^ *p* (1 − *p*)/*d* ^2^] was used to calculate the sample size (32). With 95% confidence level, proportion of medication-related burden 50% because there is no previous study in Ethiopia and relative precision 5%, the total sample size was 384. The sample size was corrected for non-response rate (15%) (33), and the final calculated sample size was 442. A systematic random sampling technique was utilized to select the study participants.

### Study variables

The dependent variable was MRB which was measured using Living with Medicines Questionnaire version 3 (LMQ-3), while the independent variables were patients’ sociodemographic variables (age, gender), type of DM, duration of DM, presence of comorbidities, number of comorbidities, adherence to the prescribed medication, number of prescribed medication, and fasting blood glucose (FBS) level.

### Data collection tools

Data were collected by structured questionnaires *via* face-to-face interviews. The questionnaire has four parts. The first section contains the participants’ sociodemographic characteristics, the second part contains items related to diseases and medication, the third part contains items related to self-reported MRB, and the fourth section contains items related to medication adherence. The LMQ-3 is a 41-item questionnaire where respondents are required to indicate their level of agreement using a five-point Likert-type scale. This tool is comprised of eight domains. The LMQ-3 tool was validated in English (34) and adapted and validated in Arabic (35). The overall LMQ score was the sum of the scores of all the 41 items in the questionnaire, with scores ranging from 41 to 205 corresponding to the following: extremely high burden, 173–205; high burden, 140–172; moderate burden, 107–139; minimal burden, 74–106; and no burden at all, 41–73. The questionnaire also contained a 10-cm line VAS scale, through which respondents provided a global assessment of the overall burden they experienced (0 “no burden at all” to 10 “extreme burden at all”), with higher scores representing greater perceived burden (16).

A 12-item ARMS rating from 1 (none of the time) to 4 (all of the time) scale ranging from 12 to 48 scores was used to measure adherence. Those patients who scored ≤13 were categorized as adherent and those who scored >13 were non-adherent (20). This tool was validated in Arabic (16) and the internal consistency was correlated to the Morisky adherence (36). Other patient-related information was then obtained from medical records using the data collection form that was specifically designed for this study.

### Data quality control

The questionnaire was translated into Amharic language and back-translated to English to check if the translated items retained the same meaning as the original items. Training was given to the data collectors by the investigator and supervisors. A pretest was done by taking 10% of the sample size to assess the understandability, internal consistency, and validity of the questionnaire. The overall LMQ-3 tool had a good internal reliability with a Cronbach’s alpha of 0.90. Each of the eight domains showed good internal consistency with Cronbach’s alpha ranging from 0.72 to 0.98. The internal consistency (Cronbach’s *α* coefficient) of the overall ARMS score was 0.828.

### Statistical analysis

Data were checked, coded, and cleaned for inconsistencies and missing values and entered into EpiData version 4.6.0.0 (EpiData Association, Odense, Denmark) statistical software and then exported to SPSS version 21 (IBM Corporation, Armonk, NY, USA) for analysis. Descriptive, correlation, comparative, and regression analyses were conducted. Continuous variables were expressed as mean ( ± SD) when normally distributed or median (IQR) when not normally distributed. Additionally, categorical variables were summarized as frequency (percentage) of the total. The normality of data was assessed by the Shapiro–Wilk test. A Spearman’s and Pearson correlation test were used to assess the relationship between the independent variables and dependent variable. Independent *t*-tests or one-way analysis of variance (ANOVA) was done to examine the MRB differences among the independent variables. Since the dependent variable MRB was a continuous variable that fulfilled the normality distribution and linearity assumptions, multiple linear regression was used to identify factors associated with MRB. Multicollinearity was assessed using Pearson correlation coefficients. In the linear regression, most of the independent variables were continuous, while a few variables were dummy categorical variables. *p*-value <0.05 was considered as statistically significant to declare association. In the study, most of the independent variables were continuous (age, duration of DM, VAS score, ARMS score, FBS, number of comorbidities), while a few variables were dummy categorical variables which were categorized as yes/no (using tablet, using injection).

## Results

### Sociodemographic characteristics of DM patients

A total of 442 participants were approached and 423 of them agreed to participate in the study giving a response rate of 95.7%. Of the participants, more than half of them (234, 55.3%) were men. The mean age of the study participants was 40.04 ( ± 15.67) years. Of the study participants, 322 (76.1%) were urban dwellers and 145 (34.3%) were unable to read and write (**Table 1**).

**Table 1:** Sociodemographic characteristics of DM patients attending at Felege Hiwot Comprehensive Specialized Hospital.

Variable	Category	Frequencies (*N*)	Percentages (%)
Sex	Male	234	55.3
	Female	189	44.7
Age (years)	18–24	68	16.1
	25–34	121	28.6
	35–44	83	19.6
	45–54	59	13.9
	55–64	52	12.3
	≥65	40	9.5
Residence	Rural	101	23.9
	Urban	322	76.1
Marital status	Married	224	53.3
	Single	142	33.6
	Divorced	21	5.0
	Widowed	36	8.5
Religion	Orthodox	325	76.8
	Muslim	84	19.9
	Protestant	14	3.3
Educational status	Unable to read and write	145	34.3
	Able to read and write	35	8.3
	Primary education	72	17.0
	Secondary education	43	10.2
	Diploma	86	20.3
	Degree and above	42	9.9
Occupational status	Housewife	58	13.7
	Student	60	14.2
	Farmer	54	12.8
	Government employee	136	32.2
	Private	99	23.4
	Other	16	3.8
Cigarette smoking	Yes	5	1.2
	No	418	98.8
Alcohol consumption	Yes	7	1.7
	No	416	98.3
Lifestyle status	Healthy diet only	46	10.9
	Exercise only	238	56.3
	Exercise and healthy diet	164	24.6
	None	35	8.3

### Clinical and medication characteristics of DM patients

Among the 423 study participants, 220 (52.0%) had type 1 DM. The median (IQR) duration of DM diagnosis was 4.0 (2.0) years. Comorbidities were present in 89 (21.0%) of the study participants, and the most commonly reported comorbidity was hypertension 65 (15.4%). Most of the participants (365, 86.3%) were prescribed with one or two medications. The median (IQR) FBS value was 198 (105) mg/dl, and 345 (81.6%) of the participants had uncontrolled DM (FBS ≥ 126 mg/dl) (**Table 2**).

**Table 2:** Clinical and medication information of DM patients attending at Felege Hiwot Comprehensive Specialized Hospital.

Variables	Category	Frequencies (*N*)	Percentages (%)
Type of DM	Type 1	220	52.0
	Type 2	203	48.0
Duration of DM	3 months–4 years	281	66.4
	Above 4 years	142	33.6
Comorbidities	Yes	89	21.0
	No	334	79.0
Type of comorbidities	Hypertension	65	15.4
	Heart failure	12	2.8
	Dyslipidemia	6	1.4
	Asthma	6	1.4
Number of prescribed medications	≤2	365	86.3
	>2	58	13.7
Medication dosage form	Injection	207	48.9
	Tablet	201	47.5
	Both injection and tablet	17	4
	Other (capsule, suspension, puff)	5	1.2
DM control status	Uncontrolled	345	81.6
	Controlled	78	18.4
Payment for prescribed medications	Yes	368	87
	No	55	13

### Medication-related burden of DM patients

The mean ( ± SD) LMQ-3 score was 126.52 (17.39). The results showed that the majority (85.1%) of DM patients suffered from a moderate (58.9%, 95% CI: 53.9–63.7) to a high (26.2%, 95% CI: 22.5–30.0) degree of burden. The mean ( ± SD) VAS score was 3.83 ( ± 1.07) and the majority of the participants (60%, 95% CI: 55–65.6) were perceived to have a high global burden (**Table 3**).

**Table 3:** Descriptive summary of perceived medication-related burden by LMQ-3 and VAS score in DM patients attending at Felege Hiwot Comprehensive Specialized Hospital.

Variable	Range	Frequency (%)	(95% CI)	Mean (SD)	Median (IQR)
LMQ overall score	41–205	–	–	126.52 (17.397)	130 (25)
Minimal burden	74–106	63 (14.9)	(11.8–18.7)	–	–
Moderate degree of burden	107–139	249 (58.9)	(53.9–63.7)	–	–
High burden	140–172	111 (26.2)	(22.5–30.0)	–	–
VAS: global burden	0–10	–	–	3.83 (1.07)	4 (2)
	Up to 4	169 (40)	(34.4–45.0)	–	–
	Above 4	254 (60)	(55–65.6)	–	–

Medication-related burden showed a moderately significant positive association with VAS score (*r* = 0.561, *p* < 0.001) and ARMS score (*r* = 0.518, *p* < 0.001). Additionally, place of residence (*r* = 0.109, *p* = 0.026), duration of DM diagnosis (*r *= 0.151, *p* = 0.002), number of comorbidities (*r* = 0.157, *p* = 0.001), and number of medications (*r* = 0.207, *p* < 0.001) had a weak positive association with MRB.

Diabetic patients with FBS ≥126 mg/dl on visit showed high MRB mean score than those with FBS ≤125 mg/dl (129.19 ± 15.9 vs. 114.68 ± 18.82, *p* < 0.001). Patients with a high VAS score ≥4 (high global burden) showed high MRB (134.04 ± 12.49 vs. 115.21 ± 17.64, *p* < 0.001) than those with a VAS score ≤3 (low burden). Additionally, patients with ARMS score ≥14 (non-adherent) showed high MRB (35.16 ± 1.86 vs. 119.47 ± 18.02, *p* < 0.001) than those with ARMS score ≤13 (adherent).

### Factors associated with medication-related burden

Simple and multiple linear regression analyses were done to identify factors associated with MRB (LMQ-3) score. Simple linear regression indicated that age, having college education, being urban, practicing a healthy diet and exercise, duration of DM, absence of comorbidities, number of comorbidities, using two or more medicines, not taking both tablet and injection, FBS level, and ARMS and VAS scores were associated with MRB. However, the multiple linear regression analysis indicated that only VAS score (*B* = 12.773, *p* = 0.001), ARMS score (*B* = 8.505, *p* = 0.001), and FBS on visit (*B* = 5.858, *p* = 0.003) were significantly associated with MRB. For every unit increase in the VAS score (global burden), ARMS score, and FBS on visit, there were 12.773, 8.505, and 5.858 increases in the MRB, respectively. The model analysis showed that the independent variables explained 37% of the variability of the dependent variable MRB (*R* ^2^ = 0.37), and the regression model was a good fit for the data (*F*_16, 404_ = 14.88, *p* < 0.001) (**Table 4**).

**Table 4:** Multiple linear regression LMQ-3 score versus sociodemographics and medicine-use characteristics in DM patients attending at Felege Hiwot Comprehensive Specialized Hospital.

Variables	*B*	Std. error	Beta	*T*	*p*-value	95% CI
Age	−0.119	0.687	0.011	−0.173	0.863	−1.469 to 1.231
Education	0.799	0.520	0.083	1.535	0.126	−0.224 to 1.822
Place of residence	−2.33	1.809	−0.057	−1.288	0.199	−5.887 to 1.227
Lifestyle	−0.308	0.792	−0.017	0.389	0.698	−1.865 to 1.249
Duration of DM (years)	−0.051	2.018	−0.001	−0.025	0.98	−4.02 to 3.92
Presence of comorbidities	−1.653	3.986	−0.039	−0.415	0.679	−9.489 to 6.183
Total number of comorbidities	1.042	1.997	0.033	0.522	0.602	−2.884 to 4.967
Presence of hypertension	−3.316	3.528	−0.069	−0.940	0.348	−10.251 to 3.619
Presence of dyslipidemia	−5.338	6.219	−0.036	−0.858	0.391	−17.562 to 6.887
Number of medications	−2.146	3.463	−0.042	−0.62	0.536	−8.953 to 4.661
Tablet	−4.424	7.317	−0.127	−0.605	0.546	−18.80 to 9.959
Injection	−2.764	7.229	−0.080	−0.382	0.702	−16.975 to 11.447
Both tablet and injection	−6.980	8.306	−0.079	−0.840	0.401	−23.308 to 9.349
Fasting blood glucose	5.858	1.958	0.131	2.992	0.003*	2.009 to 9.707
Overall perception (VAS)	12.773	1.678	0.360	7.61	0.001*	9.474 to 16.072
ARMS	8.505	1.702	0.243	4.99	0.001*	5.16 to 11.85
Constant	132.121	40.29		3.287	0.001	53.234 to 211.645

R^2^ = 0.37, adj. R^2^ = 0.345, F = 14.88, p < 0.001. * indicate statistically significant at P<0.05.

### Adherence to prescribed medication

**Figure 1:**
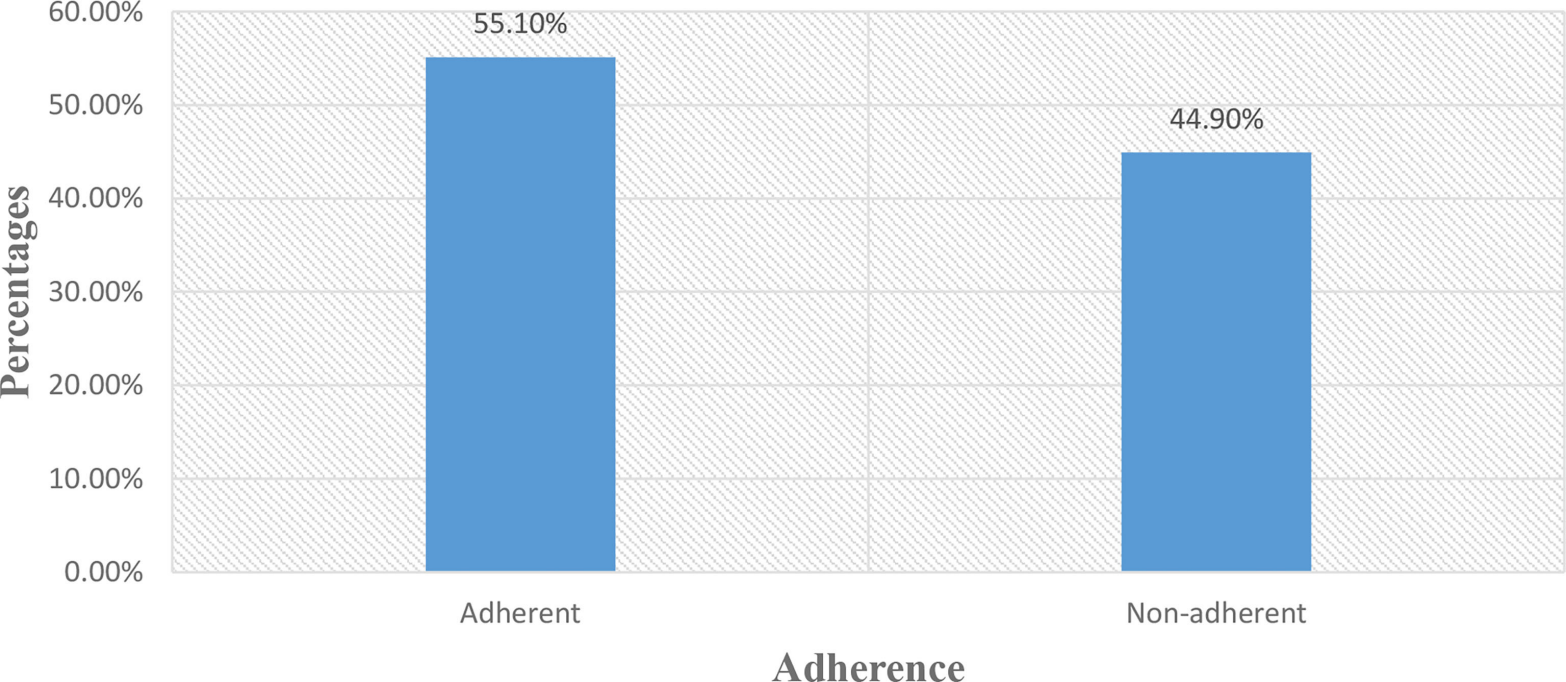
Adherence to prescribed medication among DM patients attending at Felege Hiwot Comprehensive Specialized Hospital.

The adherence to prescribed medication was measured by using the ARMS score. The median (IQR) ARMS score was 13 (3), range 12–48. The result showed that 44.9% (95% CI: 39.9–49.7) of the study participants were non-adherent to their prescribed medications and 55.1% (95% CI: 50.3–60.1) were adherent to their prescribed medications (**Figure 1**).

## Discussion

The current findings revealed that the majority of DM patients suffered from a moderate to a high degree of burden. According to the multiple linear regression, high VAS score (perceived global burden), ARMS score, and FBS on visit were significantly associated with high MRB. In the present study, almost half of the DM patients were non-adherent to their prescribed medications.

The results of the present study showed that the majority (85.1%) of DM patients suffered from moderate (58.9%, 95% CI: 53.9–63.7) to high MRB (26.2%, 95% CI: 22.5–30.0), which is similar to the findings of a study in Qatar [minimum (66.8%) to moderate (24.1%)] (16). However, these values were higher compared to the results of the studies conducted in England [minimum (33.1%) to moderate (53.6%)] (21), New Zealand [moderate (45.1) to high (30.5)] (37), and Kuwait [minimum (35.4%) to moderate (62.0%)] (20). The possible explanation for the difference could be variations in sociodemographic characteristics, presence and number of comorbidities, number of prescribed medications, duration (years) of DM diagnosis, unavailability of better medical care, poor relationship with the healthcare providers, and inadequate knowledge of patients about their medication in developing nations which might cause higher treatment burden to the current study (15, 38).

The current findings showed that the mean LMQ-3 overall score among patients ≥65 years was higher than those aged <65 years old. This finding was in line with similar studies which reported that overall LMQ scores were related to age (16, 20, 39). A possible reason might be that age was a risk factor for comorbidities, as the number of comorbidities increases the risk of polypharmacy and the complexity of medication regimens and the passive behavior of the elderly in taking their medications also increases (40–42). In contrast, a study done in England revealed a significantly lower mean LMQ score among the elderly (≥65 years) than adult patients (18–64 years) (21). The possible explanation for the difference might be variations in educational status between developed and resource-limited settings. Participants who lived in developed nations might have a better attitude and general knowledge about medicine, know about the possible side effects of medicine, and have good communication with health professionals about medicine and the perceived effectiveness of medicine which might lower MRB in elderly participants (43–45).

The MRB was positively associated with uncontrolled DM, which is similar to the study done in Qatar (16). If blood glucose increases and is not managed properly, the risk of getting acute as well as chronic complications increases, and this might cause acute life-threatening events such as diabetic ketoacidosis and increase the frequency of hospitalization and decrease the patients’ quality of life (5, 46).

In this study, MRB had a statistically significant association with non-adherence to medication. This result agrees with the studies done in Qatar (16) and Kuwait (20). A possible explanation might be that non-adherence causes a lower serum concentration of medication which affects the effectiveness and efficacy of the medication, and this causes treatment failure which might lead to uncontrolled glycemic level. Finally, it may lead to disease progression associated with acute and chronic complications, enhancing exposure to polypharmacy (19, 47). Polypharmacy obligates patients to take multiple medications at once and apply many instructions, and it also exposes the patients to possible side effects and adverse drug reactions. In addition, non-adherence leads to a poor quality of life and frequent hospitalization, and this also leads to a high MRB (48).

In the current study, 60% of the patients had a high global perceived burden. The current findings showed a moderate positive relationship with overall LMQ-3 scores (Pearson *r* = 0.561) and a weak positive correlation between VAS score and ARMS score (*r* = 0.286**)**. This implies that the higher the perceived global burden, the lower the medication adherence level.

The study had its limitations. The English version of the LMQ-3 tool was developed and used in a country with a different health system, which has not been used in Ethiopia before which may potentially limit the findings. The study does not include critically ill patients who do not regularly attend the follow-up clinic, and this might limit the representativeness of the findings.

## Conclusion

The vast majority of DM patients suffered from moderate to high degree of burden and non-adherence to long-term medicine. Adherence level, perceived global burden, and FBS were the predictors of MRB in diabetic patients. Therefore, to minimize MRB, multidimensional intervention is required for DM patients with uncontrolled DM, those non-adherent to their prescribed medications, and patients with a high perceived global burden. In addition, the findings of this study would provide data for clinicians on which factors they should target in reducing the medication-related burden of their diabetic patients.

## Data availability statement

The raw data supporting the conclusions of this article will be made available by the authors, without undue reservation.

## Ethics statement

This study was reviewed and approved by the School of Pharmacy Ethical Review Committee, College of Medicine and Health Sciences, University of Gondar. To ensure confidentiality of data, study subjects were identified using codes, and only authorized persons accessed the collected data. The patients/participants provided their written informed consent to participate in this study.

## Author contributions

AB and MB were involved in the conceptualization and design of the study and data acquisition, analysis, and interpretation and took part in drafting the initial version of the manuscript. MT and MY were responsible for the data acquisition, analysis, and interpretation and for revising and editing the initial manuscript. All authors contributed to the article and approved the submitted version.

## Conflict of interest

The authors declare that the research was conducted in the absence of any commercial or financial relationships that could be construed as a potential conflict of interest.

## Publisher’s note

All claims expressed in this article are solely those of the authors and do not necessarily represent those of their affiliated organizations, or those of the publisher, the editors and the reviewers. Any product that may be evaluated in this article, or claim that may be made by its manufacturer, is not guaranteed or endorsed by the publisher.

## Abbreviations

ARMS, Adherence to Refills and Medications Scale; DM, diabetes mellitus; FHCSH, Felege Hiwot Comprehensive Specialized Hospital; IQR, interquartile range; MRD, medication-related burden; MRB, perceived medication-related burden; SD, standard deviation; TBQ, Treatment Burden Questionnaire; VAS, visual analog scale; WHO, World Health Organization.
